# Hidden Negative Issues and Possible Solutions for Advancing the Development of High‐Energy‐Density in Lithium Batteries: A Review

**DOI:** 10.1002/advs.202401739

**Published:** 2024-04-19

**Authors:** Atsuo Yamada

**Affiliations:** ^1^ Department of Chemical System Engineering, School of Engineering The University of Tokyo Hongo 7‐3‐1, Bunkyo‐ku Tokyo 113–8656 Japan; ^2^ Sungkyunkwan University Institute of Energy Science & Technology (SIEST) Sungkyunkwan University Suwon 16419 South Korea

**Keywords:** anion intercalation, high energy density, lithium batteries, Nernst–Madelung potential, oxygen redox, salt‐concentrated electrolytes

## Abstract

This review article discusses the hidden or often overlooked negative issues of large‐capacity cathodes, high‐voltage systems, concentrated electrolytes, and reversible lithium metal electrodes in high‐energy‐density lithium batteries and provides some feasible solutions that can realize the construction of realistic rechargeable batteries with higher energy densities. Similar objective discussion of the negative aspects of lithium–air batteries, multi‐valent shuttles, anion shuttles, sulfur cathode systems, and all‐solid ceramic batteries can help fabricate more realistic batteries.

## Graphical abstract

Overviewing lithium‐based batteries, hidden or overlooked, but critical “negative” issues for higher energy density on 1) large capacity cathodes, 2) high‐voltage system, 3) concentrated electrolytes, and 4) reversible lithium metal electrode, will be briefly broiled to initiate constructive discussions toward realistic rechargeable batteries with higher energy density, pointing some important cautions for general beliefs.

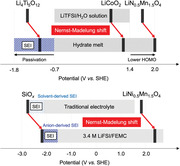

## 1 Introduction

Reports of “breakthrough” or “conceptual leaping” in battery materials and systems to solve the associated environmental issues appear nearly every day in newspapers, webpages, and journals. They are published with catchy text such as doubled, tripled, more energy density, an electric vehicle that charges in just a few minutes, definite safety, etc., with beautiful images attached. However, in many cases, these articles contain no or minimal facts and do not highlight severe technical hurdles. Such reports are hurdles for the healthy progress in battery research and development. Objectively, the negative aspects of batteries must be highlighted and addressed in the future to advance battery research and development.

This review/perspective article discusses the hidden or overlooked hurdles in the field of battery research and development so that scientists can circumvent these traps and work toward better realistic batteries. The following recent findings from our group will be described: i) energy‐inefficient square‐scheme electrochemistry of oxygen redox in cathode materials;^[^ ^1^, ^2^, ^3^, ^4^, ^5^, ^6^, ^7^ ^]^ ii) anion‐intercalation‐triggered side reactions on cathode conductive carbon additives at high potential;^[^ ^8^, ^9^ ^]^ iii) the design of multifunctional small solvent molecules as an alternative to expensive, viscous, and low‐conducting salt‐concentrated electrolytes;^[^ ^10^, ^11^ ^]^ and iv) large Nernst–Madelung potential shifts that simultaneously occur at the cathode and the anode that invalidate electrolyte optimization for a single electrode such as lithium metal.^[^ ^12^, ^13^, ^14^, ^15^ ^]^ Moreover, possible solutions are provided for each issue.

## 2 Energy‐Efficient Oxygen Redox Reaction Offer Limited Extra Capacity

The participation of oxygen in electrochemical reactions of lithium‐rich layered cathode materials increases the capacity beyond conventional cationic‐redox limits.^[^ ^16^, ^17^, ^18^, ^19^, ^20^ ^]^ O^2−^ is a regular state in oxide solid cathode materials. After the full oxidation of transition metals, O^2−^ sequentially transforms to several chemical states such as O^−^ radical, O_2_^2−^ dimer, and O_2_ molecule, upon the electrochemical oxidation of weakly p‐bonded O2p orbitals, which are formed by Li─O─Li linkages in a layered structure.^[^ ^4^, ^21^, ^22^ ^]^ Furthermore, structural transformation, typically induced by transition metal migration, makes the phenomenon complicated.^[^ ^23^, ^24^ ^]^

**Figure 1:**
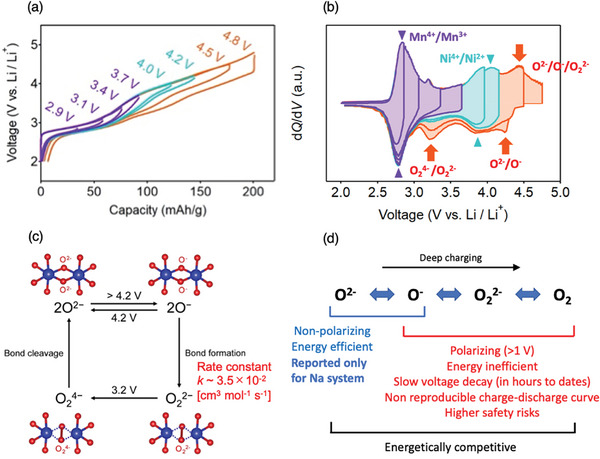
Mechanism of the extra capacity provided by oxygen redox in layer‐structured positive electrode materials. a) Galvanostatic charge/discharge curves of O2‐Li_1.12‒_ *_y_* Ni_0.17_Mn_0.71_O_2_ and b) the corresponding d*Q*/d*V* plots at C/20 with increasing upper cut‐off voltage from 2.0 to 4.8 V versus. Li/Li^+^. c) Square scheme for the non‐polarizing/polarizing oxygen‐redox reaction. The oxidation of O^2−^ to unstable O^−^ above 4.2 V versus. Li/Li^+^ leads to the formation of the stable peroxide O_2_^2−^with a rate constant of *k* = 3.5 × 10^−2^ cm^3^ mol^−1^ s^−1^, while the reduction of O_2_^2−^ to unstable O_2_^4−^ at 3.2 V versus. Li/Li^+^ induces immediate bond cleavage to O^2−^. d) Summary of the sequential oxygen oxidation upon charging and the issues with each reaction step. Only the first step of O^2−^ ↔ O^−^ is an energy‐efficient non‐polarizing redox process. (a) and (b) Reproduced with permission.^[^ ^7^ ^]^ 2022, RSC Publishing.

O2‐type stacking compounds composed of face‐shared octahedra along the stacking direction strongly prohibit transition‐metal migration because the migrated metal experiences strong cation‐cation repulsion.^[^ ^7^, ^25^, ^26^ ^]^ Hence, one can exclusively focus on the oxygen chemistry.^[^ ^7^ ^]^ A Mn and Ni‐based lithium‐rich phase, O2‐Li_1.12‒_ *_y_* Ni_0.17_Mn_0.71_O_2_, was synthesized to activate oxygen redox. Its charge–discharge curves were measured by gradually increasing the upper cutoff voltage (**Figure** **1**a). The derived dQ/dV plots provided many important implications, as shown in Figure 1b.

At an early stage of oxygen oxidation, O^2−^ transforms into an O^−^ radical, while further oxidation leads to oxygen dimerization to form O_2_^2−^. This suggests that at least two types of oxygen species co‐exist in a fully charged state: the O^−^ radical and dimerized O_2_^2−^. These two oxygen species exhibit independent reduction processes at different potentials (Figure 1b,c). The O^−^ radical is reduced back to O^2−^ in the presence of negligible polarization. However, O_2_^2−^ dimer is reduced at a much lower potential (≈3.2 V) in the presence of large polarization because dimerization is an exothermic stabilization reaction.

To summarize, four chemical species could be generated upon oxygen redox, as shown in the following square scheme reaction pathway (Figure 1c,d):

(1) $$\begin{array}{ccc} 2O^{2-} & ⇆ & 2O^{-}+2e^{-}\\ ↑ & & ↓\\ {O_{2}}^{4-} & ⇆ & {O_{2}}^{2-}+2e^{-} \end{array}$$

Notably, only the first redox process with the O^−^ radical allows non‐polarizing, energy‐efficient charge, and discharge. Even though O^−^ is primarily stabilized by multi‐orbital bond formation,^[^ ^4^ ^]^ the non‐polarizing large capacity and reversible redox reaction have been reported for the sodium intercalation system, which has a more stable layered structure than its lithium analog.^[^ ^3^, ^26^ ^]^ In addition, dimer formation (O_2_^2−^) is a very slow relaxation process ranging from hours to days, indicating that the voltage profile should be changed depending on the charge–discharge protocol and history.^[^ ^6^, ^7^ ^]^ Therefore, the practical application of oxygen redox is extremely difficult, at least in layered‐structure frameworks with 3d transition metals.

The quantitative comparison of state‐of‐the‐art Li/Mn‐rich layered oxide cathodes (≈280 mAh g^−1^ with a cut‐off charge voltage > 4.5 V) and Li‐stoichiometric Ni‐rich cathodes (≈240 mAh g^−1^ with a cut‐off charge voltage < 4.5 V) is informative. The additional capacity provided by the reversible, energy efficient oxygen redox is not much larger than the capacity observed without it. Additionally, due to the square‐scheme hysteresis and polarization arising from the sluggish kinetics of extensive oxygen redox, the Li‐rich cathodes have a lower average discharge voltage, which is worsened during cycling due to voltage fade, and a much lower energy efficiency. Consequently, while there may be a significant cost advantage to the Li/Mn‐rich cathodes, they do not provide much, if any, improvement in energy density over the Ni‐rich cathodes.

## 3 Anion Intercalation into Conductive Carbon Poisons High‐Voltage systems

When cathode materials are charged at a high potential in a cell, prior to the inherent oxidative decomposition of organic electrolytes, other side reactions usually occur at a much lower potential. Among them, anion or solvent intercalation into the conductive carbon cathode is a critical degradation mode that is often overlooked.^[^ ^8^, ^9^, ^27^, ^28^, ^29^, ^30^, ^31^, ^32^ ^]^ Anion or solvent intercalation occurs due to the very low interfacial barrier and thermodynamic stability of the intercalated phases.^[^ ^28^, ^29^ ^]^ Conductive carbon possesses a large specific surface area and can trigger side reactions even at small amounts in the electrode composite because the anion or solvent intercalation not only degrades the conductivity but also creates many defective active sites for electrolyte decomposition.^[^ ^31^, ^32^ ^]^

**Figure 2:**
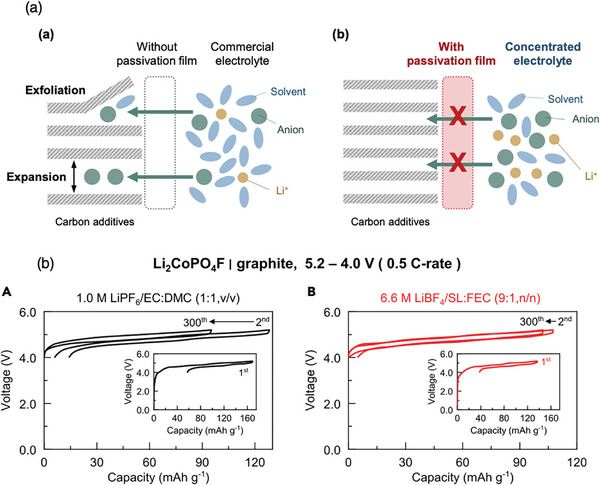
Suppression of anion or solvent intercalation into the conductive carbon of the cathode composite. a) Electrolyte design to fix and block the anion or solvent molecules. b) Charge–discharge curves of Li_2_CoPO_4_F/graphite full cells with the given electrolytes with a cut‐off voltage of 5.2–4.0 V at a constant rate (0.5 C‐rate). (b) Reproduced with permission.^[^ ^9^ ^]^ 2021, Cell Press.

A salt‐concentrated electrolyte can be used to fix anion and solvent molecules and prevent anion intercalation into the conductive carbon, as anion or solvent molecules will be strongly coordinated to lithium in a concentrated environment (**Figure** **2**a). The solvent sulfolane (SL) forms an anion‐blocking cation‐exchange cathode‐electrolyte interphase (CEI). Moreover, LiBF_4_ salt exhibits high oxidative stability and high solubility. The electrolyte composition of SL, LiBF_4_, and a small amount of fluoroethylene carbonate (FEC) (6.6 m LiBF_4_ SL/FEC (9:1, n/n)) forms a solid‐electrolyte interphase (SEI) on graphite. A cell with the above‐mentioned electrolyte, a LiCoPO_4_F cathode, and a graphite anode realized an extremely stable operation with a cut‐off voltage of 5.2 V (Figure 2b)^[^ ^9^ ^]^ and demonstrated 93% capacity retention even after 1000 cycles. However, this electrolyte showed the worst stability on Pt over 5.5 V (ref). Therefore, these two degradation modes (inherent electrolyte decomposition and anion intercalation into conductive carbon) are independent of each other.

As exemplified here, high salt concentration is a simple and effective strategy to stabilize the system under harsh electrochemical conditions. However, increasing the salt concentration is not practical in commercial applications due to the significantly higher viscosity and hence lower conductivity with an increase of materials cost. Hence, we present some alternative strategies in the following sections.

## 4 Multi‐Functional Small Solvent Molecules as Alternatives to Expensive and Viscous Concentrated Electrolytes

Increasing the salt concentration is effective because several functional combinations of salt and solvent, including the fire‐extinguishing trimethyl phosphate (TMP),^[^ ^33^, ^34^ ^]^ have been reported over the past 10 years.^[^ ^34^ ^]^ However, high‐salt‐concentration electrolytes always suffer from lower conductivity, higher viscosity, and higher weight, with significant cost‐impact, and hence do not meet industry standards. Meanwhile, the major necessity of high concentration comes from its ability to form anion‐derived functional SEI, and most reports have relied on using expensive FSI‐based salts.^[^ ^34^, ^35^, ^36^ ^]^ Thereby, the use of a diluted electrolyte with multi‐functional small solvent molecules with fire‐extinguishing and SEI formation abilities is a feasible alternative.

**Figure 3:**
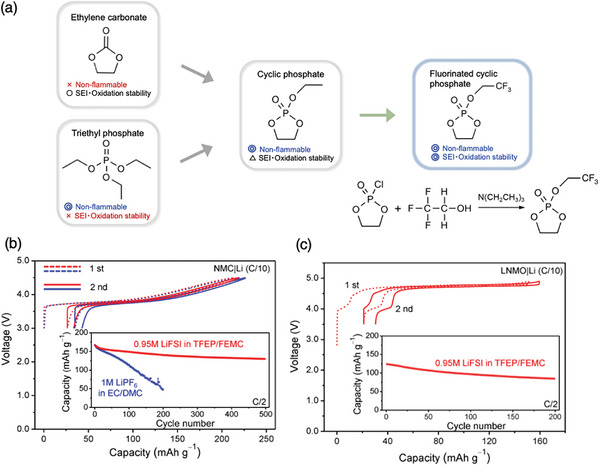
Strategies to avoid high‐concentration or solvent‐in‐salt electrolytes. a) Design of a five‐membered fluorinated cyclic phosphate, 2‐(2,2,2‐trifluoroethoxy)‐1,3,2‐dioxaphospholane 2‐oxide (TFEP), composed of a phosphate group that provides non‐flammability and a fluorine‐containing moiety that improves oxidative stability, such that it mimics the chemical structure of EC and forms a solid electrolyte interface (SEI) on the anode. b) First two charge–discharge profiles of LiNi_1/3_Mn_1/3_Co_1/3_O_2_ (NMC)|Li cells in different electrolytes at a rate of C/10. The inset shows the cycling performances of the NMC|Li cells using 0.95 m LiFSI in TFEP/2,2,2‐trifluoroethyl methyl carbonate (FEMC) and 1 m LiPF_6_ in EC/DMC at a rate of C/2 after three formation cycles at a rate of C/10 with a cut‐off voltage of 3.0–4.5 V. (c) First two charge–discharge profiles of the LiNi_0.5_Mn_1.5_O_4_ (LNMO|)Li cell in 0.95 m LiFSI in TFEP/FEMC at a rate of C/10. The inset shows the cycling performance at a rate of C/2 after three formation cycles at a rate of C/10. The cut‐off voltage was 3.5–4.9 V. All tests were conducted at 25 °C. Reproduced from Springer Nature, (b), (c). ref. [10]

A fluorinated cyclic phosphate (TFEP) solvent‐based electrolyte, composed of a cyclic carbonate that forms an SEI and a linear phosphate with fire‐retardant properties (**Figure** **3**a)^[^ ^10^ ^]^ demonstrates oxidation stability owing to the fluorine moiety, non‐flammability owing to phosphorous, and SEI and CEI formation ability with its five‐membered ring. By applying this new solvent and without using high salt concentration, very good reversibility of graphite anode was achieved, ever outperforming the LiPF_6_‐based commercial electrolyte (Figure 3b). The impact is not only limited to the anode side but also extends to the cathode side owing to the oligomeric CEI formation triggered by the surface oxygens atoms of the cathode material. The cell was stable for a higher voltage cutoff of 4.5 V for NMC and 4.8 V for the high‐voltage LiNi_0.5_Mn_1.5_O_4_ spinel. Hence, the TFEP‐based low‐concentration electrolyte significantly outperformed the conventional LiPF_6_/EC system, particularly in terms of the potential window and cycling stability, without relying on a high salt concentration.

The design and synthesis of such multi‐functional solvent molecules have been extensively explored in the past 5 years.^[^ ^37^, ^38^, ^39^, ^40^ ^]^ Another effective strategy to overcome the issues inherent to salt‐concentrated electrolytes is the use of localized high‐concentration electrolytes (LHCEs) that use a non‐solvating diluent.^[^ ^41^, ^42^, ^43^, ^44^ ^]^ LHCEs generate solvation structures dominated by contact ion pairs and aggregates, with a near‐zero amount of free solvent, while maintaining a low overall salt concentration and viscosity that is comparable to conventional electrolytes. However, even LHCEs exhibit poor conductivity and require the use of expensive fluorinated diluents.

## 5 Large Parallel “Nernst–Madelung” Potential Upshift: State‐Of‐The‐Art Electrolytes for Lithium Metal Are Unstable on the Cathode Side

**Figure 4:**
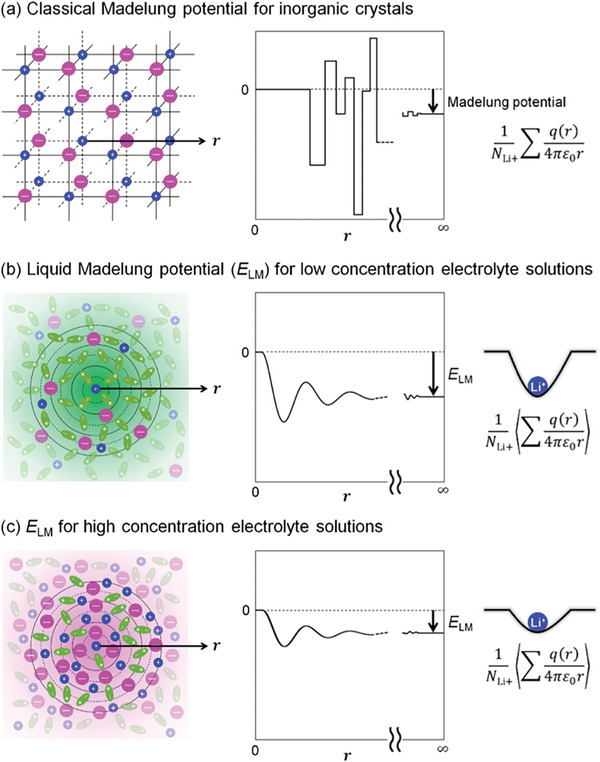
Concept of liquid Madelung potential ELM in electrolytes. *E*_LM_ represents the sum of the electrostatic interactions of the surrounding ions and/or solvents with the central Li^+^. For inorganic crystals, such a concept is known as the classical Madelung potential a), where ions are fixed in a crystal lattice, yielding discrete Coulombic energy versus. position *r* curves. However, as solvents/ions are mobile in electrolyte solutions, *E*_LM_ is calculated by both time‐ and space‐averaging the potentials from the snapshots obtained by MD simulations, resulting in a Coulombic energy versus. *r* curve with rounded features b). Li^+^ site potential was significantly shallower at higher salt concentrations c), leading to a larger potential upshift. Reproduced with permission.^[^ ^15^ ^]^ 2024, Springer Nature.

Electrode potential strongly depends on the electrolyte. The Debye–Huckel (D–H) theory^[^ ^45^ ^]^ has been used for low (diluted) salt concentration electrolytic cells since 1923. However, no suitable model exists for high salt concentration electrolytic cells, except for the extended expressions of the D–H theory based on the fitting of several parameters.^[^ ^46^ ^]^ Our group has recently proposed the concept of liquid Madelung potential (*E*_LM_),^[^ ^13^ ^]^ which provides a definite physical meaning to the activity coefficient in the Nernst equation and quantitatively explains the large shift of the electrode potential generally observed in high‐salt concentrated electrolyte cells.^[^ ^13^ ^]^ The *E*_LM_ around specific ions is analogous to that observed in solid ionic crystals and is calculated by summing all electrostatic interactions with each constituent atomic charge in surrounding solvents or ions (**Figure** **4**), where time averaging is applied to the structural information obtained by MD simulations.^[^ ^13^ ^]^

**Figure 5:**
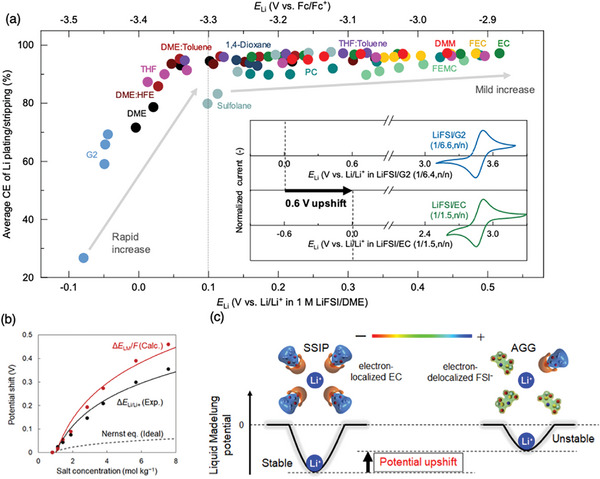
Shallow liquid Madelung potential quantitatively explains the large Li/Li^+^ potential shift and better Coulombic efficiency. a) Coulombic efficiencies (CEs) of Li plating/stripping depend on the electrode potentials of Li (*E*_Li/Li+_). The shift of *E*_Li/Li+_ (>0.6 V), which determines the CEs of Li metal anodes, strongly depends on the electrolytes. The inset shows cyclic voltammograms of ferrocene, which is the internal standard of the given electrolytes. Reproduced with permission.^[^ ^12^ ^]^ 2022, Springer Nature. b) Experimental data of Δ*E*_Li/Li+_ and the calculated potential shifts of liquid Madelung potential (Δ*E*_LM_/*F*) were designated as the shift from the data for the lowest concentrations and plotted as a function of salt concentration for LiFSI/EC. The black dashed lines represent the potential shift based on the ideal Nernst equation, wherein the Li^+^ activity coefficient (*γ*_Li+_) is unity. c) The main mechanism of potential upshift: Coulombic energy penalty caused by a change in the dominant local coordination from electron‐localized EC to electron‐delocalized FSI^−^. (b) and (c) Reproduced with permission.^[^ ^15^ ^]^ 2024, Springer Nature.

The concept was successfully applied to explain the improved Coulombic efficiency of the lithium metal electrode in (locally) concentrated electrolytes.^[^ ^15^ ^]^ Thermodynamically unstable lithium ions (shallow liquid Madelung potential) correspond to a higher deposition dissolution potential (*E_Li/Li+_*). As shown in **Figure** **5**a, upshifting *E_Li/Li+_* decreases the side reactions induced by electrolyte decomposition and improves Coulombic efficiency.^[^ ^12^ ^]^ However, the data in Figure 5a is only proof‐of‐concept collected from an electrolyte‐rich coin cell with a larger electrolyte/capacity (E/C) ratio than that of a practical cell with lean conditions (E/C = 2 g Ah^−1^).^[^ ^47^ ^]^ Therefore, careful reinvestigation is necessary to check the reversibility under a realistic battery condition with lean electrolytes.

Another important outcome from Figure 5a is the large variation range of *E_Li/Li+_* (>0.6 V). The calculated *E*_LM_ (i.e., Δ*E*_LM_/*F*, divided by the Faraday constant for unit conversion from [eV] to [V]) versus *m*_Li+_ reasonably reproduces the concentration dependence of the experimental upshift of Δ*E*_Li/Li+_ as shown in Figure 5b. The shallow liquid Madelung potential in concentrated electrolytes is primarily attributed to the electrostatic interactions of the first coordination sphere, which are dominated by anions rather than solvent molecules. Li^+^ is electrostatically more stable when Li^+^ is strongly solvated by electron‐localized oxygen atoms in EC solvents, whereas it is unstable when coordinated by the electron‐delocalized FSI^−^ (Figure 5c).

The rational concept of “liquid Madelung potential” provides a long awaited “quantitative” explanation for the potential shift in high salt‐concentrated regions that largely exceeds the concentration limit of the Debye–Huckel theory established one century ago in 1923,^[^ ^45^ ^]^ thus providing a firm physical meaning to the activity coefficient in the Nernst equation.

**Figure 6:**
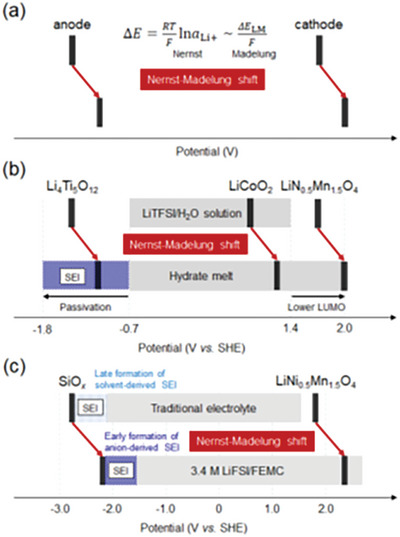
Schematic of the parallel “electrolyte‐dependent” Nernst–Madelung electrode potential shift and apparently “electrolyte‐independent” constant cell voltage. a) The overall general trend of the Nernst equation and liquid Madelung potential *ΔE*_LM_, where *a*_Li+_ is the activity of Li^+^. (b,c) Typical examples for aqueous and nonaqueous systems, along with simultaneous modulation of the thermodynamic electrolyte stability window and the characteristics of the solid‐electrolyte interphase (SEI). Reproduced with permission.^[^ ^49^ ^]^ 2023, the Electrochemical Society of Japan.

According to the Nernst equation, an increasing activity coefficient suggests a large shift in the electrode potential at both the anode and the cathode in a battery system. **Figure** **6** shows the typical electrolyte‐dependent potential diagrams for Li_4_Ti_5_O_12_/LiNi_0.5_Mn_1.5_O_4_ and SiO_x_/LiNi_0.5_Mn_1.5_O_4_ cells.^[^ ^14^, ^48^, ^49^ ^]^ The diagrams illustrate that the observed output voltage of the cell is independent of the electrolyte, leading, in many cases, to overlook the huge intrinsic “Nernst–Madelung shift” that occurs at both the cathode and the anode. However, anode stabilization may destabilize the cathode when the anodic stabilization limit of the electrolyte window is reached or exceeded. Particularly, many state‐of‐the‐art electrolytes optimized to stabilize lithium metal should be carefully reexamined because they may not provide suitable conditions for the stable operation of standard NMC‐based commercial high‐voltage cathodes. Notably, excessive upshifting of electrode potential leads to cycling failure of the LiNi_0.8_Mn_0.1_Co_0.1_O_2_|Li system while improving the reversibility of the Li metal itself.^[^ ^50^ ^]^ In this context, using a low voltage cathode such as LiFePO_4_ may result in stable lithium metal batteries. Alternatively, overall tuning and optimization of the Madelung shifts of the electrodes and the potential window of the electrolyte can change the total potential landscape and SEI formation, as demonstrated by the successful SiO_x_/LiNi_0.5_Mn_1.5_O_4_ system.^[^ ^14^ ^]^

## 6 Concluding Remarks

Several negative rationales, that could help realize better realistic, high‐energy‐density lithium batteries, have been overlooked in the field of battery research and development. In this review/perspective article, we described the following cautionary aspects against the conventional beliefs held in the field of battery research:

- Energy‐efficient oxygen redox reactions in layered positive electrode materials can offer only limited extra capacity.
- Anion intercalation into cathode conductive carbons poisons high‐voltage battery systems.
- As the battery industry does not want to adopt salt‐concentrated electrolytes, multi‐functional small solvent molecules can be a feasible alternative.
- State‐of‐the‐art electrolytes for lithium metal can be unstable at the cathode side due to a large parallel “Nernst–Madelung” potential upshift.

We also provided some feasible solutions for each issue. We believe that similar objective investigations into the negative issues with exotic batteries such as lithium–air batteries, multi‐valent shuttles, anion shuttles, sulfur cathode systems, and all‐solid ceramic batteries could help fabricate more realistic batteries.

## Conflict of Interest

The authors declare no conflict of interest.
